# Impact of HPV educational intervention on knowledge and vaccination intentions among Ukrainian migrant and refugee parents in Poland

**DOI:** 10.3389/fpubh.2025.1647569

**Published:** 2025-09-26

**Authors:** Maria Ganczak, Paweł Kalinowski, Marta Kowalska-Babiak, Mufida Nazrieva, Serhij Nyankovskyy, Michael Edelstein

**Affiliations:** ^1^Department of Infectious Diseases, Collegium Medicum, University of Zielona Gora, Zielona Góra, Poland; ^2^Department of Hygiene and Epidemiology, Faculty of Health Sciences, Medical University of Lublin, Lublin, Poland; ^3^Medical Center “Medyk”, Rzeszow, Poland; ^4^Department of Pediatrics 1, Danylo Halytsky Lviv National Medical University, Lviv, Ukraine; ^5^Azrieli Faculty of Medicine, Bar-Ilan University, Ramat Gan, Israel

**Keywords:** HPV, knowledge, vaccination, intent, Ukraine, migrants, intervention

## Abstract

**Background:**

Despite the availability of HPV vaccines, uptake remains low among Ukrainian adolescents. Educational interventions can enhance parents’ knowledge and intent regarding HPV vaccination.

**Objective:**

To evaluate the effects of a prospective, culturally tailored, evidence-based HPV vaccine face-to-face educational intervention on knowledge and vaccine intent among Ukrainian migrant and refugee (UMR) parents in Poland, utilizing a pre-post design.

**Methods:**

A study was conducted among 178 UMR parents between February and July 2024. Using telephone calls, research staff recruited consecutive parents registered to the network of primary care clinics “Medyk” in Rzeszów, Poland. Eligible parents were those having children aged 9–17 years, who had not completed the HPV vaccination. They completed pre- and post-data on HPV knowledge and intent. Four female Ukrainian GPs were trained to deliver 9 group interventions (2 h each); this had to be changed to 27 individual 1-h sessions after an erroneous suspicion of HPV vaccine adverse effects, which spread out in the Ukrainian community and resulted in a recruiting crisis. Data were analyzed using McNemar’s test and multilevel regression analysis.

**Results:**

The majority of participants were female (84.3%) and aged >30 years (77.5%); 59.6% of UMR parents presented a low knowledge level (≤50%). Only 30.3% knew all possible routes of HPV transmission, and 39.9% knew male HPV-related neoplasms. The intervention significantly improved HPV knowledge by 63.4% (pre: 5.2, SD ± 2.1; post: 8.2, SD ± 1.7; *p* < 0.0001). Parents who attended individual education had lower pre-intervention scores but outperformed parents who participated in the group sessions in post-intervention knowledge. Ukrainian mothers and parents with higher SES showed a significant improvement in vaccine intent after the intervention, from 56.6 to 64.8%, *p* = 0.04, and from 55.3 to 73.9%, *p* = 0.046, respectively.

**Conclusion:**

The study finds that educational interventions for UMR parents can improve their understanding of HPV and support informed vaccination decisions for their children. The integration of specific approaches—such as culturally sensitive messaging, the utilization of trained Ukrainian presenters, and tailored health literacy strategies based on the community needs—may provide critical support for future implementation efforts.

## 1 Introduction

Poland has a significant Ukrainian minority, with migration escalating since the start of the Russia-Ukraine war (1). Before the 2022 conflict, approximately 1.25 million Ukrainians resided in Poland as economic migrants (2, 3). However, after February 2022, approximately 17.3 million people crossed the Polish-Ukrainian border in search of safety, resulting in a substantial demographic shift (4).

Providing access to healthcare for refugees was a significant public health challenge. The financial impact was substantial, with Poland facing the highest healthcare and social costs among nations hosting Ukrainian refugees, estimated at around 8.4 billion euros in 2022 (5). Ukrainians who crossed the border after February 24, 2022, gained access to comprehensive healthcare, including preventive vaccinations and other essential services. For instance, all Ukrainian refugees have free access to COVID-19 vaccinations. Currently, children residing in Poland for at least 3 months are required to be vaccinated against 11 infectious diseases, as outlined in the national immunization program (NIP) (3, 6, 7). These vaccinations are provided free of charge and are administered in primary healthcare centers, just like for Polish citizens (8). As part of this effort, a cost-free HPV vaccination was introduced into the Polish NIP in 2023 for children aged 9 to 18. It is important to note that HPV vaccination has not yet been included in Ukraine’s National Vaccination Schedule and is only available privately (9).

Notably, prophylactic HPV vaccines have been developed to prevent infections and diseases, as well as to decrease mortality associated with the most common oncogenic types. These vaccines offer protection against high-risk HPV types, which are responsible for approximately 70 to 90% of cervical cancers, along with a substantial share of other HPV-related cancers (10). In Poland, the highest rates of HPV-associated cancers among women are observed in both invasive and *in situ* cervical cancer. In 2020, the incidence was approximately 10 cases, equating to 5 cases per 100,000 women. Cervical cancer ranks as the sixth leading cause of cancer overall and is the third most common cancer among women aged 15 to 44. For men, the most common HPV-related cancer is laryngeal cancer, with about 7 cases per 100,000 men diagnosed in 2020. Each year, around 4,400 women and 1,600 men in Poland are diagnosed with diseases that could be effectively prevented through HPV (11).

Despite the efforts outlined, the Ukrainian community in Poland continues to be under-vaccinated due to a variety of barriers common to immigrant communities. These barriers include issues such as population mobility and instability of residence, along with a lack of awareness and knowledge about vaccines, misconceptions regarding their efficacy and safety, mistrust in healthcare and vaccines, and insufficient strong recommendations from healthcare providers (6, 12, 13). A systematic review of studies examining the factors encouraging HPV vaccine uptake among teenagers identified several personal factors. These include having a greater knowledge of the vaccine, relying on healthcare providers for information, and maintaining a positive attitude toward vaccines, all of which contribute to higher vaccination rates (14). Therefore, interventions for improving knowledge and fostering positive attitudes toward the HPV vaccine may help increase vaccination coverage. However, several factors are anticipated to pose limitations or challenges in implementing such interventions, particularly among minority populations (15). These reported obstacles can be grouped into several categories, including prior negative experiences with vaccination in individuals’ countries of origin, societal norms, cultural differences, and systemic barriers within the healthcare system. Such barriers may include language constraints exacerbated by a lack of professional interpreting services, issues related to insurance status, and logistical challenges such as transportation difficulties, appointment scheduling issues, and the need for additional office visits (16–18).

In prior qualitative research we conducted with Ukrainian migrant parents in Poland (13), it was found that they prefer to receive information about HPV vaccination in their native language and in small group settings within healthcare environments. They believe that healthcare providers can play a crucial role in promoting HPV vaccination. Similar preferences have been expressed by other minority groups in various contexts (19–22).

Based on these findings, we created an interactive educational intervention for Ukrainian migrant and refugee (UMR) parents. A four-component intervention was implemented, including outreach, education, navigation to services, and provision of a free HPV vaccine series for eligible Ukrainian children and adolescents. The approach was based on the effectiveness of these elements in a previous program that evaluated a culturally tailored, evidence-based educational intervention designed to improve HPV vaccine completion among a predominantly low-income, migrant population (20). The intervention was adapted to meet local needs. In this study, we aimed to assess the impact of this intervention on the knowledge and intentions of UMR parents to vaccinate their children against HPV, encompassing both the outreach and education components. This initiative was part of the larger European RIVER-EU project, which aims to enhance vaccination rates among children and adolescents in underserved communities by addressing systemic barriers across four European countries, including Poland (13, 23).

## 2 Materials and methods

### 2.1 Study design

The intervention consisted of four components decribed above. This paper reports on the first two components of the intervention, namely outreach and education, as described below. The third component, navigation, was delivered by program navigators (one migrant health coordinator and two receptionists). They provided Ukrainian parents with vaccination scheduling assistance and also performed tracking and reminder services. Reminder phone calls were made one and 2 weeks after the initial attempt. After the unsuccessful phone contact attempt, no additional effort was made to contact the participants. Access to the vaccine was provided after the education was delivered. Eligibility criteria for receiving a no-cost HPV vaccine through our program included being aged 9–18 years and being insured. For those eligible for the no-cost vaccine, a certified nurse administered the vaccine at the recruitment site; this was the provision component of the intervention. Participants were continually recruited throughout the study period, though the study was limited to the first 180 participants due to the time required to complete the project’s work package.

### 2.2 Setting and recruitment

The intervention took place from February until July 2024, in Rzeszów, the capital of the Podkarpackie province in southeastern Poland, which borders Ukraine. This province has the highest number of border crossings with Ukraine (24). Participants were consecutively recruited from the database of patients registered at the *Medyk* Medical Center in Rzeszów. They were invited to participate in the study by two Ukrainian receptionists through telephone calls.

The *Medyk* Medical Center provides both commercial and services financed by the National Health Fund. As part of primary health care, the facility serves over 55,000 patients. Currently, *Medyk* operates an extensive network of 35 primary healthcare facilities, located in Rzeszów and the Podkarpackie province. On March 23, 2022, a special surgery dedicated to refugees from Ukraine was opened under the clinic network in response to the humanitarian crisis caused by the outbreak of war. Its patient population reflects the broader Ukrainian refugee population in Poland in terms of age, gender, and socio-economic status (SES). A clinic is staffed by qualified medical personnel from Ukraine, who speak the native language of patients. Since the clinic’s inception, approximately 8,000 patients from Ukraine have registered for a family doctor. Of this group, more than 3,000 individuals are children and adolescents aged 0 to 18 years.

### 2.3 Eligibility

Eligible participants for the project were parents or guardians of children aged 9 to 17 who had not yet completed the HPV vaccine series. To receive a free HPV vaccine for their child, participants needed to be insured.

### 2.4 Intervention

This educational intervention was developed based on findings from focus groups and meetings within the Ukrainian migrant community that highlighted barriers to vaccination and knowledge gaps in healthcare (12, 13). These insights informed our adaptation of a previously published HPV vaccination intervention (20). Following suggestions from Ukrainian parents, we recruited four Ukrainian GPs in Poland for this initiative, compensating them for their contributions. On February 26, 2024, these GPs, together with 44 other Ukrainian healthcare professionals, participated in a 5-h online training session led by vaccination experts. After training, the Ukrainian GPs collaborated with our research team to culturally adapt and translate educational materials from the National Institute of Public Health. We also developed a PowerPoint presentation covering HPV transmission, HPV-related cancers, and the HPV vaccines—their indications, regulations, schedule, effectiveness, and potential side effects. Over nine educational sessions held every Saturday, bilingual GPs presented the material in Ukrainian, using audiovisual aids. This approach effectively increased knowledge in migrant communities (20). Each 2-h session was designed to create an inviting atmosphere, with refreshments provided and research team supervision ensuring a meaningful experience for all participants.

At the beginning of the educational sessions, the objectives of the RIVER-EU project were outlined, and participants provided informed consent before completing a comprehensive questionnaire. This self-administered, anonymous questionnaire in the Ukrainian language was used as the data collection instrument. It was developed following a thorough literature review (14, 19, 20). After the investigators designed the questionnaire, face validity was confirmed by four experts in family medicine, pediatrics, public health, and infectious diseases. They assessed and validated the instrument, providing several suggested modifications to improve the content and clarity. Filling out the questionnaire took approximately 10–15 min. The pre-post questionnaire consisted of 21 questions, divided into three parts: demographic data (age, gender, residency in Ukraine, education, SES, marital status, and time spent in Poland), knowledge about HPV, and intention to vaccinate a child against HPV. Ten multiple-choice knowledge questions were divided into three sections: regarding transmission routes, clinical manifestations, and the HPV vaccinations. Then, to measure the internal consistency of the questionnaire and to detect any flaws in the survey, it was piloted on eight Ukrainian mothers.

On May 9, 2024, a 13-year-old participant received his first dose of the Gardasil vaccine but was later diagnosed with viral meningitis, resulting in a six-day hospitalization. Investigations confirmed there were no adverse events following immunization. Nevertheless, news of the hospitalization spread through the Ukrainian community, causing a dramatic decline in parental participation in the project and registration for HPV vaccinations. The research team met immediately online with four Ukrainian GPs who delivered educational sessions. The training approach was modified by providing HPV education during parents’ clinical visits at their GPs’ offices. Eligible parents were informed of the opportunity for individual educational sessions led by their Ukrainian GP, following similar protocols to group sessions. Another adjustment made to the session was reducing its duration due to the work overload of GPs. While the timeframe for the PowerPoint presentation and fulfilling questionnaires remained unchanged, the allotted time for questions was consequently reduced.

### 2.5 Measures

Demographic information was collected on various factors, including age, gender, marital status, number of children, self-assessed SES, residence in Ukraine, length of residence in Poland, and the intention to vaccinate a child against HPV (responses were categorized as yes, no, or I do not know). A set of ten questions was administered to measure knowledge levels. Each correct response was assigned one point, indicating that higher total scores reflected greater knowledge.

### 2.6 Statistical analysis

Data were checked for completeness and internal consistency before analysis in Statistica 13.3 (TIBCO Software Inc., 2017). Continuous variables are reported as mean (SD), and categorical variables are reported as n (%). The primary outcome was the total HPV knowledge score. Within each socio-demographic stratum, pre- and post-intervention scores were compared using paired t-tests after checking distributional assumptions with the Shapiro–Wilk test. For dichotomous outcomes (e.g., parents’ willingness to vaccinate and item-level correctness), pre–post changes were evaluated using McNemar’s test. Between-group differences in the post-intervention score were assessed using ANCOVA with the baseline score centered at the sample mean (PRE_c) as a covariate and the following fixed factors: intervention type (group vs. individual), sex, age category (≤30 vs. > 30 years), marital status, SES, and prior intention to vaccinate. Type III sums of squares were used. Adjusted means (estimated marginal means; EMMeans) with 95% confidence intervals were reported. The homogeneity-of-slopes assumption was examined by adding PRE_c × factor interactions; when a significant interaction was detected, effects were interpreted conditionally at PRE = mean (i.e., PRE_c = 0), and simple effects were presented as needed. Effect sizes are presented as partial η^2^. Change scores were defined as *Δ* = (post − pre) and summarized with 95% CIs for each subgroup. Cluster-adjusted inference on Δ was performed, accounting for clustering by intervention type, using linear mixed models with a random intercept for intervention type. Given that only two clusters were available, these cluster-adjusted results were interpreted with caution. Differences between category levels were also estimated from multilevel linear models of the post-intervention score adjusted for baseline and all covariates; coefficients (*β*), standard errors, and *p*-values are reported. All tests were two-sided with *α* = 0.05.

## 3 Results

A total of 180 Ukrainian parents participated. Responses were obtained from 98.9% of participants; two questionnaires were excluded due to incomplete data. Table 1 presents the socio-demographic characteristics of the participants. The majority were female (84.3%) and over 30 years old (77.5%), having arrived in Poland 3 years ago or less (95.4%); more than half were residents of cities with more than 100,000 inhabitants in Ukraine (54.6%); and 88.8% described their SES as average or higher. Regarding the type of education provided during our intervention, 52.8% of UMR parents participated in group training, while the rest received individual training.

**Table 1:** Socio-demographic and other data of study participants.

Variable		*n*	%
Gender	Female	150	84.3
	Male	28	15.7
Age category	≤20	24	13.5
	21–30	16	9.0
	31–40	67	37.6
	>40	71	39.9
Marital status	Do not want to answer	31	17.4
	In relationship	131	73.6
	Single	16	9.0
Residency	Village	36	20.2
	City: up to 250,000 inhabitants	45	25.3
	250,001-500,000	28	15.7
	>500,000	69	38.8
Time of stay in Poland	Up to 1 year	20	11.2
	1–3 years	150	84.2
	>3 years	8	4.6
SES	Very good	40	22.5
	Average	118	66.3
	Poor	20	11.2
Type of educational session	Group	94	52.8
	Individual	84	47.2

Rzeszów, Poland, 2024; *N* = 178.

### 3.1 HPV knowledge scores

Before the intervention, 59.6% of respondents answered fewer than 50% of HPV knowledge questions correctly, 25.8% answered between 50 and 80%, and 14.6% answered more than 80% correctly. Table 2 provides details about the responses. Specifically, before the intervention, less than one-third of UMR parents were aware of all possible routes of HPV transmission, and 39.9% correctly identified male HPV-related neoplasms. Only 24.2% understood why HPV vaccination is primarily recommended for children aged 9–13, and 39.9% recognized a true statement about immunity following HPV vaccination. The percentage of correct responses to all 10 knowledge questions significantly increased after the intervention (Table 2).

**Table 2:** Correct answers to HPV knowledge questions among Ukrainian parents pre- and post-educational intervention, Rzeszów, Poland, 2024; *N* = 178.

Statement	Correct answers Pre-intervention		Correct answers Post-intervention		χ^2^_McN_	*p*
	*n*	%	*n*	%		
HPV is the name of a group of viruses	116	65.2	147	82.6	15.3	0.0001
The possible routes of HPV include…	54	30.3	159	89.3	97.4	<0.0001
Cancers and other diseases related to HPV infection in men include anal cancer, penile cancer, head and neck cancers, and genital warts	71	39.9	142	79.8	53.9	<0.0001
In Poland, girls and boys aged 12–13 years are entitled to cost-free HPV vaccination	116	65.2	161	90.5	31.7	<0.0001
HPV vaccination in Poland is non-mandatory	104	58.4	165	92.7	50.7	<0.0001
Two HPV vaccines are registered and available in Poland free of charge	120	67.4	166	93.3	40.5	<0.0001
Immunity after vaccination lasts longer than after natural HPV infection	70	39.3	90	50.6	5.8	0.02
HPV vaccination is recommended primarily for children aged 9–13 because t**he** post-vaccination response is much better than in older age groups	43	24.2	113	63.5	52.9	<0.0001
Factors that increase the chances of developing cervical cancer include …	133	74.7	165	92.7	24.0	<0.0001
Scientific studies have not confirmed a risk that vaccinated teens will start sexual contact earlier	98	55.1	151	84.8	40.4	<0.0001

**Figure 1:**
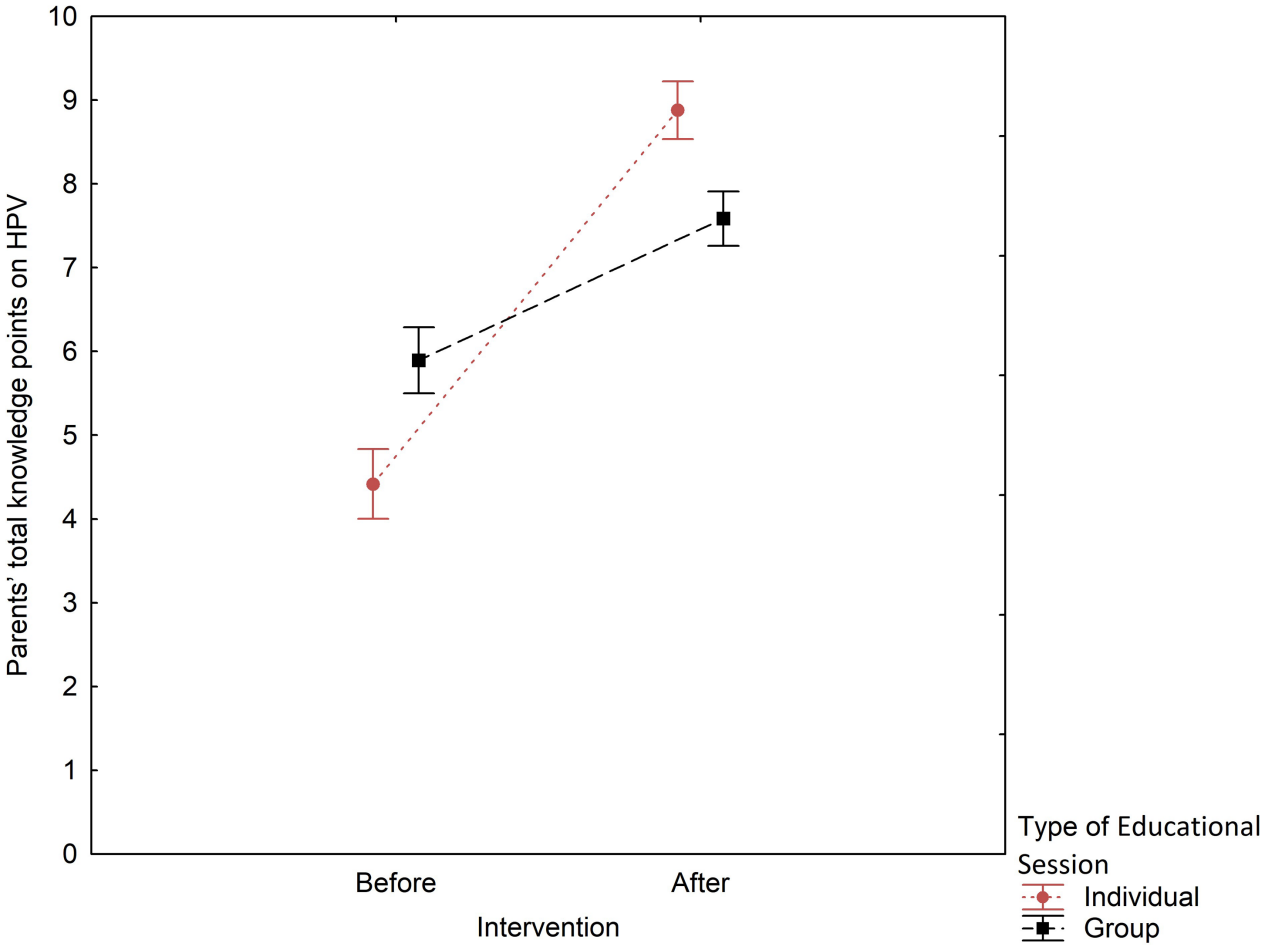
Group vs. individual educational sessions by total knowledge scores pre- and post-intervention. Rzeszów, Poland, 2024; *N* = 178.

For all parents combined, mean knowledge scores improved with the intervention, indicating a relative increase of 63.4% (*p* < 0.0001; Table 3). Concerning the type of educational intervention, a significant increase in knowledge was observed in both groups. However, those attending group training initially demonstrated a higher level of HPV knowledge compared to participants in individual sessions (5.9 ± 1.9 vs. 4.4 ± 2.0, *p* < 0.0001). Additionally, individual training resulted in a significantly greater average increase in knowledge compared to group training (8.9 ± 1.5 vs. 7.6 ± 1.7, *p* < 0.001). Parents who attended individual education sessions had lower pre-intervention scores; however, they out-performed parents in group sessions in post-intervention knowledge (Figure 1). Furthermore, having the intention to vaccinate before the intervention was associated with a greater impact of the intervention on HPV knowledge scores (*p* = 0.002).

**Table 3:** Overall knowledge scores pre- and post- HPV intervention by selected variables, Rzeszów, Poland, 2024; *N* = 178*.

Variable	Unadjusted Scores					*P*-Values before multilevel Analysis*	Estimated Pre–post differences in scores. Confidence intervals		P-values for change from Pre- to Post-Intervention (from Multi-level Model)*	Regression coefficients. Standard errors and *p-*values for category differences		
		Pre-intervention		Post-intervention								
	*n*	*M*	SD	*M*	SD		Difference (SE)	95% CI		Coefficient	SE	*p*
Total. N	178	5.2	2.1	8.2	1.7	<0.0001	3.0 (0.2)	2.6–3.4	<0.0001	–	–	–
Type of educational intervention												
Group	94	5.9	1.9	7.6	1.7	<0.0001	1.7 (0.2)	1.3–2.1	-	−1.37	0.26	<0.0001
Individual	84	4.4	2.0	8.9	1.5	<0.0001	4.5 (0.3)	4.0–5.0	-	-	-	-
Gender												
F	150	5.2	2.1	8.2	1.6	<0.0001	3.0 (0.2)	2.6–3.4	<0.0001	0.19	0.33	0.56
M	28	5.4	2.1	8.3	2.1	<0.0001	2.9 (0.6)	1.7–4.1	0.06	-	-	-
Age category												
≤30 years	40	4.8	2.1	8.3	1.6	<0.0001	3.5 (0.4)	2.8–4.2	0.0003	0.43	0.31	0.17
>30 years	138	5.3	2.1	8.2	1.8	<0.0001	2.9 (0.2)	2.4–3.3	<0.0001	–	–	–
Marital status												
In relationship	131	5.2	2.2	8.3	1.6	<0.0001	3.1 (0.2)	2.6–3.5	<0.0001	0.39	0.30	0.19
Others	47	5.2	1.6	7.9	2.0	<0.0001	2.8 (0.4)	2.1–3.5	<0.0001	–	–	–
SES												
Average and bad	138	4.9	2.0	8.4	1.7	<0.0001	3.4 (0.21)	3.0–3.8	<0.0001	0.48	0.30	0.11
Very good	40	6.1	2.0	7.7	1.6	0.0005	1.55 (0.41)	0.7–2.4	0.03	–	–	–
Intent to vaccinate before intervention												
Yes	94	5.0	2.2	7.8	1.8	<0.0001	2.8 (0.28)	2.3–3.4	<0.0001	−0.74	0.24	0.002
No/No idea	83	5.5	1.8	8.7	1.6	<0.0001	3.2 (0.28)	2.7–3.8	<0.0001	–	–	–

*Results of multilevel regression, which adjusts for sampling design effect with clustering of parents at the level of an intervention type. Regression coefficients, standard errors, and *p*-values are the results of the association tests between the post-score and the variable of concern, adjusted for all other variables in the table and the baseline score (multilevel model). A positive regression coefficient means the second category is superior, whereas a negative coefficient indicates that the first category is superior.

### 3.2 Intent to vaccinate

In ANCOVA (Type III) adjusting for baseline (PRE), there were two significant main effects—intervention type (*p* < 0.001) and prior intention to vaccinate (*p* = 0.003)—and a trend for SES (*p* = 0.070); other factors were not significant (Table 4). Notably, there was one significant interaction with the covariate: PRE_c × intervention type (*F* = 7.26, *p* < 0.001, η^2^ ≈ 0.08), implying non-parallel slopes. Therefore, EMMeans are reported and interpreted at PRE = mean (centered baseline).

**Table 4:** Pre/post descriptive results and ANCOVA-adjusted estimates.

Factor	*N*	Pre M (SD)	Post M (SD)	*p*- value (paired)	EM Means (SE) **#**	95% CL **#**	*p*-value ANCOVA	η^2^
Total, N	178	5.20 (2.06)	8.20 (1.72)	<0.0001	–	–	–	
Type of Educational Intervention on HPV Vaccination								
Group	94	5.89 (1.91)	7.59 (1.71)	<0.0001	7.3 (0.21)	6.88–7.72	<0.001	0.14
Individual	84	4.42 (1.96)	8.88 (1.45)	<0.0001	8.65 (0.25)	8.16–9.15		
Gender								
W	150	5.16 (2.07)	8.17 (1.64)	<0.0001	8.05 (0.19)	7.67–8.44	0.646	<0.01
M	28	5.39 (2.06)	8.32 (2.13)	<0.0001	7.9 (0.3)	7.31–8.49		
Age Category								
≤30 years	40	4.80 (2.08)	8.30 (1.62)	<0.0001	8.17 (0.27)	7.65–8.69	0.212	0.01
>30 years	138	5.31 (2.05)	8.17 (1.75)	<0.0001	7.78 (0.23)	7.33–8.23		
Marital Status								
In relationship	131	5.21 (2.20)	8.29 (1.61)	<0.0001	8.15 (0.21)	7.74–8.56	0.244	0.01
Others (ref.)	47	5.15 (1.63)	7.94 (1.97)	<0.0001	7.8 (0.27)	7.26–8.34		
SES								
Average and poor	138	4.93 (2.01)	8.36 (1.71)	<0.0001	8.24 (0.18)	7.88–8.6	0.070	0.02
Very good (ref.)	40	6.10 (2.02)	7.65 (1.64)	0.0005	7.71 (0.29)	7.13–8.28		
Intent to vaccinate before intervention								
Yes	94	4.98 (2.24)	7.79 (1.75)	<0.0001	7.63 (0.23)	7.17–8.08	0.003	0.05
No/No idea (ref.)	83	5.46 (1.84)	8.66 (1.58)	<0.0001	8.33 (0.22)	7.89–8.76		

Paired tests compare raw pre vs post within strata. ^**#**^ANCOVA columns are adjusted at PRE = mean; paste EMMeans (adjusted means) and 95% CI from GLM/EMMeans output. p (ANCOVA main effect) corresponds to the two-level contrast for each factor.

EMMeans for the group intervention participants was 7.30 ± SE = 0.21, and for the individual education participants, 8.65 ± 0.25 points, respectively.

Furthermore, having the intention to vaccinate pre-intervention was associated with a greater impact of the intervention on HPV knowledge scores. Individuals declaring a willingness to be vaccinated had a statistically significantly lower mean EMMean score of 7.63 ± 0.23 compared to the remaining participants, 8.33 ± 0.22 points (*p* = 0.003).

In Table 5—a continuation of the layout introduced in Table 4—each column summarizes the pre–post difference (*Δ*) in knowledge test scores attributable to the educational intervention. The individual-session subgroup exhibited the most significant mean improvement (Δ = 4.46 points; 95% CI, 3.95–4.98), while the most minor mean change was noted in the ‘very good’ SES subgroup (Δ = 1.55 points; 95% CI, 0.72–2.38).

**Table 5:** Raw Δ, cluster-adjusted p on Δ, and multilevel model coefficients.

Category	*N*	Δ (SE) [simple]	95% CI [simple]	*p* value after multilevel (cluster-adjusted on Δ)#	β (SE) [multilevel adjusted]†	*p-value* for differences
Total, *N*	178	3,00 (0,20)	2,61-3,39	<0.0001	–	–
Type of educational intervention on HPV vaccination						
Group	94	1.69 (0.21)	1.27–2.11	–	−1.37 (0.26)	< 0.001
Individual (ref.)	84	4,46 (0.26)	3.95–4.98	–	–	–
Gender						
W	150	3.01 (0.21)	2.61–3.42	<0.0001	0,19 (0.33)	0.560
M (ref.)	28	2.93 (0.58)	1.74–4.12	0.0611		–
Age category						
≤30 years	40	3.50 (0.35)	2.78–4.22	0.0003	0.43 (0.31)	0.168
>30 years (ref.)	138	2.86 (0.23)	2.40–3.31	<0.0001		–
Marital status						
In relationship	131	3.08 (0.23)	2.62–3.54	<0.0001	0.39 (0.3)	0.191
Others (ref.)	47	2.79 (0.35)	2.07–3.50	<0.0001		–
SES						
Average and bad	138	3.42 (0.21)	3.01–3.83	<0.0001	0.48 (0,30)	0.107
Very good (ref.)	40	1.55 (0.41)	0.72–2.38	0.0259		–
Intent to vaccinate before intervention						
Yes	94	2.81 (0.28)	2.26–3.36	<0.0001	−0.74 (0.24)	0.002
No/No idea (ref.)	83	3.20 (0.28)	2.65–3.76	<0.0001		–
ICC	–	–	–	–	0.44	–

Notes. ‘Δ (SE) [simple]’ and ‘95% CI [simple]’ are unadjusted pre–post differences. ‘p after multilevel (cluster-adjusted on Δ)’ are p-values from random-intercept models fitted to Δ within each subgroup #Linear Regression with clustering effect; β (SE) [multilevel adjusted]’ and ‘p difference’ come from the multilevel regression adjusting for other variables (†Linear Regression adjusted for all other variables in the table and for the baseline score). A positive regression coefficient means the second category is superior, whereas a negative coefficient indicates that first category is superior.

In all subgroups except men, the change in Δ remained statistically significant after accounting for clustering; among men, the trend was positive but did not reach significance (cluster-adjusted *p* > 0.05), consistent with the smaller sample size and the conservative behavior of cluster adjustment when only two clusters are available (Table 5).

Differences between category levels were assessed with a multilevel linear model (*β* column), adjusting for baseline score and all other covariates. In agreement with the ANCOVA results, intervention type and prior intention to vaccinate were significant: outcomes favored individual sessions over group sessions, and absence of previous intent was associated with higher adjusted post-intervention scores, consistent with larger gains in knowledge.

Pre-intervention, 56.6% of mothers expressed willingness to vaccinate a child for HPV, compared to 37.0% of fathers; the difference was not significant (p > 0.05). Intent increased post-intervention to 64.8% among mothers and decreased to 33.3% among fathers (*p* = 0.002). In the group of 92 parents who declared their intent to vaccinate a child against HPV before the educational session, 9.8% changed their minds after the session (Table 6). Conversely, among the 80 parents who initially did not express a willingness to do so, 25.0% changed their minds. In all cases, the number of participants who changed their minds about vaccination to positive after the training was higher than those who shifted their intent from positive to negative (except for the group of men, where one father changed his mind to negative and no one changed to positive). Despite the observed trend (with more than twice as many parents changing from “No/No idea” to “Yes” as vice versa), the difference in proportions of parents was not significant (*p* = 0.06). In females (*p* = 0.04) and parents who self-assessed their SES as very high (*p* = 0.046), the intent to vaccinate a child for HPV increased significantly after the intervention.

**Table 6:** Intent to vaccinate a child against HPV before and after the educational intervention.

Category	*N*	Willingness to vaccinate a child for HPV				*χ* ^2^_McN_	*p*
		Pre-intervention		Post-intervention			
		*n*	%	*n*	%		
Total	172	92	53.5	103	59.9	3.45	0.063
Gender							
Female	145	82	56.6	94	64.8	4.32	0.038
Male	27	10	37.0	9	33.3	<0.01	≈1.00*
Age Category							
≤30 Years	36	19	52.8	23	63.9	1.13	0.289
>30 Years	136	73	53.7	80	58.8	1.71	0.19
Marital Status							
In Relationship	129	73	56.6	80	62.02%	1.89	0.169
Other	43	19	44.2	23	53.6	0.90	0.343
SES							
Average / Bad	134	71	53.0	75	56.0	0.45	0.502
Very Good	38	21	55.3	28	73.9	4.00	0.046
Type of Intervention							
Group	92	52	56.6	59	64.1	1.71	0.190
Individual	80	40	50.0	44	55.0	1.13	0.289
Level of knowledge before intervention (score)							
≤5.2	101	55	54.0	62	61.4	2.11	0.146
>5.2	71	37	52.1	41	57.8	0.75	0.386

Rzeszów, Poland, 2024; *N* = 178. *Change from “yes” to “no” after intervention.

### 3.3 Post-intervention knowledge by change in the intent to vaccinate a child for HPV

Table 7 cross-tabulates the change in knowledge (decrease/no change/increase) against the change in intention to vaccinate (decrease/no change/increase) following the intervention. In the subgroup with decreased knowledge (*n* = 14), no parent reported an increase in vaccination intent (0/14). In the no-change group (*n* = 26), 15.4% showed increased intent (4/26), and in the knowledge-increase group (*n* = 132), 12.1% showed increased intent (16/132). The proportion reporting a decrease in intent was 7.1, 7.7, and 4.6% in the decline, no-change, and increase knowledge groups, respectively.

**Table 7:** Change in parents’ post-intervention knowledge by change in the intent to vaccinate a child for HPV.

Change in HPV knowledge post-intervention	Change in the intent to vaccinate a child against HPV post-intervention				χ^2^	*P*
	Decrease	No change	Increase	Total		
Decrease (n)	1	13	0	14	2.77	0.597
% row	7.1%	92.9%	0.0			
% of total	0.6%	7.6%	0.0	8.1%		
No change (n)	2	20	4	26		
% row	7.7%	76.9%	15.4%			
% of total	1.2%	11.6%	2.3%	15.1%		
Increase (n)	6	110	16	132		
% row	4.6%	83.3%	12.1%			
% of total	3.5%	64.0%	9.3%	76.7%		
Total	9	143	20	172		
%	5.2%	83.1%	11.6%	100.0%		

Rzeszów, Poland, 2024; *n* = 172. Knowledge change was computed as post-pre and categorized as decrease (< 0), no change (= 0), or increase (> 0). The shift in intention was derived from paired pre- and post-responses and coded as a decrease, no change, or increase.

A global test of association between the two 3-level variables was not significant (Pearson’s *χ*^2^(4) = 2.77, *p* = 0.60). These results should be interpreted cautiously, given several small expected cell counts.

## 4 Discussion

### 4.1 Results overview

The baseline survey conducted before the intervention indicated that UMR parents possessed inadequate knowledge regarding HPV and exhibited a low intention to vaccinate their children. The intervention, implemented by Ukrainian medical personnel in Poland, significantly enhanced overall HPV knowledge, particularly among parents who initially demonstrated a higher intent to vaccinate and those who participated in individual sessions. Despite the observed positive trend in the intention to vaccinate children against HPV before and after the intervention, the overall proportions of UMR parents did not change significantly. Nevertheless, a marked increase in willingness to vaccinate was observed among female participants and parents with higher SES following the intervention.

### 4.2 Knowledge

To the best of the authors’ knowledge, this study represents the first community-based investigation focusing on the impact of a multicomponent educational intervention regarding the HPV vaccine for children of UMR residing in a Polish province adjacent to Ukraine. Before the intervention, most UMR parents exhibited limited knowledge of HPV; however, their knowledge significantly improved following the intervention. These results align with findings from a US study involving parents of Hispanic-origin children aged 9–17 who had not completed the HPV vaccine series (20). The increase in knowledge can likely be attributed to the dual approach implemented in our intervention, which combined PowerPoint presentations with face-to-face discussions between Ukrainian parents and GPs. Additionally, educational brochures and pamphlets in Ukrainian were provided to support understanding. While some studies employed a passive approach by solely offering booklets and educational materials (25), others adopted a more active strategy that included live presentations (26, 27). Research indicates that passive methods are generally less effective in enhancing knowledge or vaccination intent (28, 29). Moreover, several studies have demonstrated that bilingual HPV education can yield significant improvements in knowledge and intention to vaccinate among vulnerable populations (20, 28, 30, 31).

Our study revealed that an intervention involving a standard PowerPoint presentation, followed by an interactive discussion regarding HPV disease and vaccination, significantly improved parents’ knowledge. This outcome highlights the effectiveness of a conventional educational strategy in enhancing parental awareness of the HPV vaccine, a finding that aligns with previous research (27, 32).

We used a structured PowerPoint presentation, but the presenter plays a crucial role in engaging UMR parents in discussions about HPV vaccination. A scoping review highlights that physicians play a key role in informing and recommending vaccinations to parents (33). During the initial phase of our intervention, Ukrainian GPs in Poland identified gaps in their knowledge about the HPV vaccine. Therefore, it’s vital to train healthcare professionals to improve their understanding and communication skills regarding the vaccine.

At baseline, nearly two-thirds of Ukrainian parents recognized that HPV refers to a virus, and three-quarters were aware of factors that elevate the risk of developing cervical cancer. However, only approximately one-third accurately identified the transmission routes of HPV and the potential for HPV-related neoplasms in males. Additionally, a significant knowledge gap regarding the HPV vaccine was reported among these parents. This limited knowledge may be attributed to insufficient educational initiatives in Ukraine, specifically within primary care centers, institutions, and the media. Furthermore, the emphasis on essential mandatory vaccinations outlined in the NIP (6, 34, 35) could lead to diminished interest among parents in expanding their knowledge about HPV and the HPV vaccine. The optional nature of the HPV vaccine may further contribute to a lack of conviction regarding its benefits.

An assessment of knowledge regarding HPV revealed that while most UMR parents have limited awareness of HPV and the vaccine, they possess a notable understanding of vaccination regulations in Poland, including the eligible population and the optional nature of the vaccination. This suggests that these parents may consult government resources to inform their decisions on vaccinating their children. In contrast, a 2019 study among medical students in Ukraine indicated that significantly more respondents were aware of HPV-related cervical cancer than of the HPV vaccination itself (35). A subsequent study published 4 years later found that 31% of them were not knowledgeable about the HPV vaccine, and 30% were uncertain whether vaccination was mandatory or included in the NIP (34). These findings suggest that awareness of HPV among the general Ukrainian population may be even lower.

The findings revealed that parents who participated in individual education sessions initially had lower pre-intervention knowledge scores. However, they exhibited greater improvements in knowledge compared to parents who participated in group sessions after the intervention. The possible explanation of this finding could be that the two groups were formed under different circumstances (proactive sign-up vs. opportunistic recruitment during a clinical visit) and were not randomized. The first group was recruited through telephone invitations, which allowed them several days to prepare and broaden their understanding of HPV. This advance notice may have attracted more health-conscious individuals than those who attended the individual sessions, often spur-of-the-moment visits to a GP. Consequently, while the baseline knowledge of HPV among group participants was relatively higher, the increase in their knowledge was less significant. This phenomenon may suggest that pre-existing knowledge could hinder the assimilation of new information (36). Therefore, the observed differences in outcomes could be due to these fundamental differences in the participants’ baseline characteristics and motivations, rather than the format of the intervention itself. As this may be due to selection effects, further research is needed to compare the effectiveness of these formats.

Furthermore, our findings suggest that the intervention may be more effective in enhancing HPV knowledge among parents who initially intended to vaccinate their child against HPV. The connection between knowledge and vaccination intention is not uniform (37–39). Generally, greater knowledge leads to increased awareness, which is typically linked to a higher intention to vaccinate. Nevertheless, our results, like others (37–40), indicate that this trend may not hold universally; a substantial increase in HPV knowledge did not translate into a significant rise in Ukrainian parents’ vaccination intent overall. Such a result underscores the need for a possible shift in focus from imparting knowledge to facilitating health behavior change. Notably, the Health Belief Model suggests that knowledge is only one component; perceived susceptibility, severity, benefits, and barriers are also critical (41). Our intervention may have increased knowledge (addressing perceived benefits) but failed to overcome key barriers (e.g., safety concerns, mistrust), which were amplified by the coincidental meningitis case. As such, implementing HPV educational interventions for UMR, like ours, could move beyond education and knowledge acquisition and smoothly orient towards the application of established, well-validated behavior change theories that have successfully guided similar health behavior interventions (42).

Interestingly, parents with less knowledge about HPV displayed a marginally higher initial intention to vaccinate, a trend that persisted post-intervention. Moreover, among the group that saw an increase in knowledge after the intervention, the percentage of parents intending to vaccinate their child increased slightly, compared to the group where knowledge remained unchanged. Our findings also revealed that enhancing knowledge does not always correlate with an increased vaccination intent; notably, one in five parents who improved their knowledge after the intervention decreased their vaccine intent. These insights shed light on a complex and nuanced relationship between HPV vaccine knowledge and vaccination intent, which contrasts with broader research findings.

A systematic review on this topic has indicated a strong correlation between the two in the general population (39). In the context of migrants, especially among the UMR population, merely providing more information might not significantly boost parents’ intentions to vaccinate their child against HPV, particularly among fathers. Hence, interventions aimed at improving knowledge and targeting Ukrainian parents must expand beyond conventional knowledge-sharing techniques and communication strategies to effectively increase HPV vaccination intent regarding their children.

### 4.3 Intent to vaccinate

Numerous interventions targeting parents have identified HPV vaccine intention as a primary outcome, paralleling the focus of our study (28). Previous research has established that parents require comprehensive information regarding HPV to mitigate misunderstandings and enable informed decision-making concerning the acceptance or refusal of the HPV vaccination (32, 43, 44). In our study, a positive trend was observed among UMR parents concerning their intention to vaccinate children for HPV, both before and following the intervention. However, the intervention did not yield a statistically significant increase in overall parental willingness to vaccinate, indicating that considerable work remains to be accomplished. This somewhat disappointing outcome may stem from the timing of our assessment of vaccination intent, which occurred immediately after the intervention, potentially limiting parents’ opportunities to reassess their attitudes. Some educational studies have documented significant improvements in vaccination intention when evaluations were conducted shortly after the intervention; however, these studies predominantly focused on adolescents and young adults rather than parents (32).

Notably, the shift from planned 2-h group sessions to 1-h individual sessions represents a significant change in the intervention’s dose and format, which might have impacted the outcomes. Although the content delivered in the 1-h individual session was identical to the 2-h group session, the group sessions may have facilitated peer-to-peer discussion and social norming, which was lost in the face-to-face format. Moreover, regarding individual sessions, Ukrainian parents were recruited spontaneously while registering for a regular visit to the GP’s office. Therefore, they might be less willing to participate in a study and less interested in HPV vaccination for their children. These are crucial differences that may partly explain the limited impact on vaccination intent.

Parental differences may influence a parent’s willingness to vaccinate their children (45). In our study, female gender was associated with a greater likelihood of intending to vaccinate children against HPV, both before and following our intervention. This counterintuitive finding is striking and warrants a more thorough discussion. This outcome is consistent with the work of Oka et al., which demonstrated that Japanese mothers tend to display a more positive attitude toward vaccinating their children for HPV compared to fathers (46). A systematic literature review and meta-analysis of global parental acceptance regarding HPV vaccinations for their children revealed that mothers are more willing to get their daughters vaccinated (47).

In the context of the UMR population, merely providing more information might not significantly boost parents’ intentions, particularly among fathers. Such disparity may be attributed to entrenched gender roles in Ukraine, where men are often regarded as primary providers and women assume responsibility for household duties and childcare (48). Research has shown that Ukrainian mothers play an essential role in the vaccine decision-making process for their children, and fathers frequently exhibit neutrality or indecision concerning HPV vaccination. These traditional gender norms likely influenced observed differences in vaccination intentions between fathers and mothers. Presumably, the educational intervention, by detailing HPV-related cancers and possible vaccine side effects, actually increased anxiety or perceived risks among fathers, who may have had a lower baseline engagement with child health matters. Specifically, the group sessions may have facilitated peer-to-peer discussions, which could raise concerns among male participants, even if the session moderator tried to minimize these worries. As participants became more informed, they might be better able to identify potential obstacles that they had not previously considered. Furthermore, our study has not explored the gender of children of fathers who reported less intention to vaccinate against HPV after the intervention. Notably, according to medical literature, parental acceptance regarding HPV vaccinations for their children was higher for daughters than for sons (47).

Differences between mothers and fathers highlight the importance of targeted educational efforts about the HPV vaccine for children. Research suggests that the intention to vaccinate may mediate the relationship between psychosocial factors and actual vaccination rates (48). Follow-up measures to assess vaccine uptake among Ukrainian migrant and refugee children are ongoing, with results to be reported in a future study.

Additionally, it was determined that parents of higher SES demonstrated a significantly greater willingness to vaccinate their children against HPV, a correlation that has also been reported by other authors (49–51).

The association between the vaccination intent and uptake presents mixed evidence. A meta-analysis revealed a positive correlation between parents’ intentions and HPV vaccine uptake (52). Conversely, other studies found that intentions do not significantly influence uptake, particularly in multivariable analyses; various factors appear to weaken this relationship (53). Some researchers argue that merely educating patients does not effectively increase vaccination rates, highlighting the “information deficit” fallacy — the mistaken assumption that vaccine hesitancy is strictly due to a lack of information (54); a “one and done” strategy, education alone will not translate into behavior change (42, 54, 55). While knowledge is a necessary first step, it is not always sufficient to counterbalance behavioral change; it is rather an essential component of a multifaceted intervention (54, 56). Therefore, a shift in focus from imparting knowledge to facilitating health behavior change is urgently needed (42, 55).

A layered approach of interventions alongside education is needed to address poor practices. This helps explain why male Ukrainian refugees’ intention to immunize their children for HPV has continued to fall below expected rates, despite educational efforts and available vaccines. Some individuals, particularly fathers, still oppose HPV vaccinations for children regardless of the information provided.

The study presents hypothetical situations where fathers reported less intention to vaccinate their child against HPV after our educational intervention. While intention is a relevant proximal outcome, it is not a reliable predictor of actual vaccination uptake. The relationship between parental knowledge and child vaccination in real-life settings is unclear, underscoring the need for further research.

### 4.4 Limitations

One of the study’s significant limitations is that we employed a pragmatic design, which did not include a parallel control group to establish the exact effect of the intervention on vaccination intent; this reduced the capacity to draw causal inferences. This limitation arose from constraints related to project timelines and budgetary restrictions, which ultimately resulted in insufficient staffing. Furthermore, the study was conducted exclusively within a network of primary care centers (PCCs) in a single Polish city. As a significant number of parents from other PCCs, potentially residing in various Polish provinces, were excluded, the generalizability of the findings is limited to this specific context.

Studies have shown that individuals who volunteer for public health interventions tend to be better educated, have higher SES, and lead more active lives than those who do not. Additionally, individuals who are personally interested in a specific topic are more likely to participate in a research study about it (57). Presumably, the broader population of Ukrainian parents in Poland presents lower HPV knowledge and intention to vaccinate a child than reported in our study. Therefore, self-selection into the intervention (especially regarding the group format) likely biased outcomes.

The measure used for “intent to vaccinate” is a simple categorical variable (Yes/No/I do not know). However, this construct is more complex. Therefore, future studies should use a more robust scale, such as a Likert scale measuring strength of intention or readiness to vaccinate (e.g., the 5-point scale used in many Health Belief Model studies); this would provide more nuanced data than a simple binary choice.

Moreover, the expressed intent of parents to vaccinate their children was speculative, thus raising questions about whether they would manifest their stated intentions in practice. Analyzing data confined to within-group changes restricts the ability to determine whether improvements in HPV vaccination intent were attributable to the educational intervention or influenced by social desirability bias, as participants may have inferred the study’s objectives (28). In addition, although intention is an essential proximal predictor of behavior, it does not always translate into action. The study was based on a brief, single-session intervention, and the assessment was restricted to immediate changes in knowledge and self-reported intent to vaccinate, without detecting possible long-term retention or sustained changes.

Public health campaigns about HPV in Poland could have influenced the results. Indeed, the health communication strategy for the national HPV vaccination program, which included media campaigns, billboards, leaflets, and web marketing, was initiated in June 2023, concurrent with the program’s launch (8). However, our study began in March 2024, 9 months after the national program had started, and the pre-intervention knowledge level for the whole group was alarmingly low. Therefore, the observed changes in parents’ HPV knowledge are likely attributable to the intervention, rather than being due to a simple temporal association.

## 5 Conclusions and recommendations

This investigation holds significant potential for public health advancements, particularly in diminishing HPV-related morbidity and mortality rates. By fostering increased vaccination coverage among at-risk populations, particularly those represented by UMR, our study aims to make a meaningful contribution to the overall well-being and health outcomes of these communities.

The study finds that educational interventions for UMR parents can improve their understanding of HPV and support informed vaccination decisions for their children. Structured programs, whether group or individual, effectively enhance knowledge, but increased understanding does not always translate to a higher intention to vaccinate.

More broadly, our findings can significantly inform educational interventions designed to increase vaccination uptake among migrants and refugees. First, this intervention emphasized the need to build upon previous research. In our case, we successfully assessed the barriers and facilitators to vaccine deployment in this vulnerable community (12, 13, 48). Focus group discussions with Ukrainian parents indicated that involving Ukrainian GPs and health promoters – the most trustworthy HCWs - could enhance educational initiatives and improve parental willingness to vaccinate their children against HPV. Moreover, to address language barriers, it was crucial to hire Ukrainian healthcare workers for educational presentations and to provide informative materials in the Ukrainian language. All the aforementioned opinions of Ukrainian community members were then essential for understanding the implementation, adaptation, and customization process (29, 56). Notably, systematic reviews conducted by Smulian et al. (58) and Escoffery et al. (29) have highlighted significant gaps, with fewer than 20% of studies reporting facilitators and only 30% addressing barriers. Additionally, our Ukrainian experts emphasized the need for training medical staff in vaccine knowledge and communication skills to enhance understanding and motivation. In conclusion, future interventions should be tailored to meet the unique needs of underrepresented Ukrainian parents.

Secondly, our intervention has demonstrated that implementing a previously established, evidence-based intervention within migrant populations (20) remains an effective strategy. The ultimate design of such an intervention can be refined through pertinent adaptations derived from collaborative discussions among experts, community members, and stakeholders.

Research indicates that educational efforts are most effective when implemented at multiple levels (29). However, Ukrainian healthcare professionals need to present vaccination information clearly and in a structured manner. Following the presentation, engaging activities like summarizing and gathering feedback can further enhance understanding (59).

Barriers to vaccine intent, particularly HPV vaccine hesitancy among Ukrainian fathers, must be addressed through culturally tailored educational programs delivered by trained Ukrainian professionals. Health events can significantly impact vaccination perceptions in migrant communities; for instance, a child’s hospitalization for viral meningitis, shortly after receiving the HPV vaccine, led to decreased confidence among UMR parents. A similar incident in Ukraine in 2008, where a student’s death during a measles-mumps-rubella vaccination campaign resulted in dramatically lower vaccine uptake, highlights the need for prompt and effective responses to adverse events (60). Maintaining community trust in vaccinations requires an objective investigation of such incidents. Continuous monitoring of interventions will help enable timely responses to address challenges. Further studies, primarily randomized controlled trials, are needed to assess long-term effectiveness.

## Data Availability

The raw data supporting the conclusions of this article will be made available by the authors, without undue reservation.
